# Nuclear import of RNA polymerase II is coupled with nucleocytoplasmic shuttling of the RNA polymerase II-associated protein 2

**DOI:** 10.1093/nar/gkt455

**Published:** 2013-05-30

**Authors:** Diane Forget, Andrée-Anne Lacombe, Philippe Cloutier, Mathieu Lavallée-Adam, Mathieu Blanchette, Benoit Coulombe

**Affiliations:** ^1^Institut de Recherches Cliniques de Montréal (IRCM), Montréal, Québec, Canada H2W 1R7, ^2^McGill Centre for Bioinformatics and School of Computer Science, McGill University, Montréal, Québec, Canada H3A 2B4 and ^3^Department of Biochemistry, Université de Montréal, Montréal, Québec, Canada H3T 1J4

## Abstract

The RNA polymerase II (RNAP II)-associated protein (RPAP) 2 has been discovered through its association with various subunits of RNAP II in affinity purification coupled with mass spectrometry experiments. Here, we show that RPAP2 is a mainly cytoplasmic protein that shuttles between the cytoplasm and the nucleus. RPAP2 shuttling is tightly coupled with nuclear import of RNAP II, as RPAP2 silencing provokes abnormal accumulation of RNAP II in the cytoplasmic space. Most notably, RPAP4/GPN1 silencing provokes the retention of RPAP2 in the nucleus. Our results support a model in which RPAP2 enters the nucleus in association with RNAP II and returns to the cytoplasm in association with the GTPase GPN1/RPAP4. Although binding of RNAP II to RPAP2 is mediated by an N-terminal domain (amino acids 1–170) that contains a nuclear retention domain, and binding of RPAP4/GPN1 to RPAP2 occurs through a C-terminal domain (amino acids 156–612) that has a dominant cytoplasmic localization domain. In conjunction with previously published data, our results have important implications, as they indicate that RPAP2 controls gene expression by two distinct mechanisms, one that targets RNAP II activity during transcription and the other that controls availability of RNAP II in the nucleus.

## INTRODUCTION

Synthesis of mRNAs and many snRNAs by the eukaryotic enzyme RNA polymerase II (RNAP II) is central to proper cell function. Many factors and mechanisms that regulate transcription by RNAP II have been identified and characterized [reviewed in (1–8)]. A number of these factors are recruited to the RNAP II transcription complex via interactions with the carboxyl terminal domain (CTD) of its largest subunit, POLR2A/RPB1, a domain made of a heptapeptide repeated 52 times in humans (9–12). The function of the CTD is extensively regulated by phosphorylation and is not limited to transcriptional regulation, as the coupling of transcription with pre-mRNA processing involves binding of regulatory factors to the CTD (13–15).

The molecular process by which the 12-subunit RNAP II enzyme is built before transcription has not been extensively studied, partly because of the lack of knowledge on the protein machinery involved in this process. Recently, characterization of the network of interactions for RNAP II in the soluble cell fraction identified many factors that participate in the process of RNAP II biogenesis [(16–19); see (20) for a recent review]. RPAP4/GPN1 is an essential conserved member of a newly discovered family of GTPases that share a unique Gly-Pro-Asn (GPN) loop motif (21–23). Forget *et al.* (17) have shown that silencing, overexpression or nuclear sequestration of RPAP4/GPN1 after leptomycin B (LMB) treatment results in the cytoplasmic accumulation of the two largest subunits of RNAPII, POLR2A/RPB1 and POLR2B/RPB2. Additional physical interaction and functional data indicate that RPAP4/GPN1 plays a role in coupling RNAP II nuclear import to the process of microtubule assembly (17). The role of RPAP4/GPN1 in RNAP II nuclear import has been confirmed and further detailed by other reports (24,25). Notably, Bertrand and colleagues (26) reported that the chaperone HSP90 and its cofactor RPAP3 are also involved in nuclear import of human RNAP II through a mechanism that requires pre-assembly of RNAP II subunits in the cytoplasm. The co-chaperone RPAP3 is part of a recently characterized complex (27,28) that is tightly connected to RNAP II subunits and the other RPAPs (29). Cramer and colleagues (30) have shown that the protein Iwr1 binds to RNAP II and regulates nuclear import of the enzyme in yeast. The role of the human homolog of Iwr1, SLC7A6OS, in nuclear import of RNAP II remains to be established.

The RNAP II-associated protein 2 (RPAP2) is a central component of the RNAP II interaction network, defined using protein affinity purification coupled with mass spectrometry (AP–MS) from the soluble cell fraction (19). Examination of the amino acid sequence of RPAP2 revealed the presence of a zinc-finger and Rtr1-homology domain. Indeed, yeast has two genes showing homology with *RPAP2*, namely, *Rtr1* and *Rtr2* (31). Two recent articles have reported that human *RPAP2* and yeast *Rtr1* encode a phosphatase that specifically removes the phosphate at Ser5 of the CTD of the largest RNAP II subunit POLR2A/RPB1 (32,33). Although one report showed that Rtr1/RPAP2 participates in the transition from Ser5 to Ser2 during transcription, the other report proposed that RPAP2 specifically targets transcription of snRNA genes. Detailed mechanisms of RPAP2 regulation remain to be characterized. No function has yet been reported for *Rtr2*. Rtr1 and Rtr2 are not essential for yeast cell growth.

In this article, we report on the function and mechanism of RPAP2 in human cells. Our results indicate that RPAP2 is a mainly cytoplasmic protein that shuttles between the cytoplasm and the nucleus. They further reveal that an RPAP2 N-terminal fragment spanning amino acids 1–170 supports nuclear localization of RPAP2 and interacts physically with RNAP II. A RPAP2 C-terminal fragment spanning amino acids 156–612 specifies cytoplasmic localization and underlies direct interaction with the GTPase GPN1/RPAP4, which has previously been shown to also shuttle between the cytoplasm and the nucleus while being involved in nuclear import of RNAP II. Most notably, our results indicate that silencing of GPN1/RPAP4 not only interferes with RNAP II nuclear import as we described previously but also blocks RPAP2 nuclear export. We propose a model in which the CTD phosphatase RPAP2 can regulate transcription in two distinct ways, by catalyzing CTD dephosphorylation during transcription and regulating RNAP II availability in the nuclear space.

## MATERIALS AND METHODS

### Antibodies

The antibodies used in this study were obtained from various sources: unphosphorylated RNA pol II monoclonal antibody (8WG16) (Covance), anti-FLAG monoclonal antibody (Sigma), anti-phospho-Ser/Thr-Pro clone CC3 (Cederlane), RNA Pol II H5 monoclonal antibody (Covance), RNA Pol II H14 monoclonal antibody (Covance), Pol II (N20) polyclonal antibody (Santa Cruz Biotechnology), histone H3 monoclonal antibody (Upstate), anti-RPAP2 antibody (Proteintech Group), anti-MBDin/XAB1 (DP-17) polyclonal (Sigma), CDK9 (C-20) polyclonal antibody (Santa Cruz), B-tubulin monoclonal antibody (clone TUB 2.1), horseradish peroxidase-conjugated secondary antibody (GE Healthcare), Cy3 dye (Cederlane) and Alexa Fluor 488 (Invitrogen).

### Cytoplasmic, nuclear and chromatin extracts

Preparation of the cytoplasmic and nuclear fractions was performed using the nuclear extraction kit from Active Motif. For chromatin extraction, the pellet obtained after nuclear fractionation was resuspended in buffer containing 3 mM ethylenediaminetetraacetic acid, 0.2 mM EGTA, 1 mM DTT and protease inhibitor cocktail. The resuspended pellet was sonicated twice for 15 s using a Sonic Dismembrator model 100 (Fisher Scientific). For western blotting analysis, 8 μg of each fraction was loaded on gels.

### Transfection, siRNA silencing and pharmacological inhibition of CRM1 by leptomycin B

Transfection experiments for generating stable or transient HeLa cell lines expressing FLAG-tagged versions of RPAP2 and POLR2A used lipofectamine, as described by the supplier (Life Technology). RPAP4/GPN1 (ON-TARGETplus SMART pool), RPAP2 (ON-TARGETplus SMART pool) and control (siCONTROL non-targeting pool) siRNAs (Dharmacon) were doubly transfected into HeLa cells using oligofectamine (Invitrogen) at siRNA final concentration of 100 nM (17). The efficiency of silencing was monitored for each experiment using western blotting. Pharmacological inhibition of the CRM1/NES nuclear export pathway was achieved using LMB (10 ng/ml).

### Immunofluorescence and imaging

Immunofluorescence and imaging using HeLa cells were performed as previously described (17).

### *In vitro* GST pull-down

For *in vitro* pull-downs, GST and His-tagged proteins were purified as described by the supplier (GE Healthcare and Qiagen, respectively). Ten picomoles of the GST protein and 1 pmol of the His protein or highly purified calf tymus RNAP II were pre-incubated in binding buffer for 1 h at 4°C, before adding 25 µl of glutathione–Sepharose beads as previously reported (34).

### Co-immunoprecipitation assays

Cells were transiently or stably transfected with expression vectors in 60-mm dishes and lysed by adding 250 µl of lysis buffer containing 50 mM Tris–HCl, pH 7.5, 120 or 250 mM NaCl, 5 mM ethylenediaminetetraacetic acid, 0.3% CHAPS or 0.5% NP40, 1 mM NaF, 0.5 mM Na–orthovanadate, 1 mM DTT, 0.1 mM PMSF, 1 µg/ml leupeptine, 1 µg/ml aprotinin and 1 µg/ml pepstatin. The lysis solution was incubated at 4°C for 30 min with agitation, mixed with 5 µl of anti-FLAG M2 Affigel (Sigma) and incubated at 4°C for 2 h with agitation. The beads were washed four times with lysis buffer before being processed for sodium dodecyl sulfate gel analysis.

## RESULTS

### RPAP2 is a mainly cytoplasmic protein that shuttles between the cytoplasm and the nucleus

**Figure 1.**
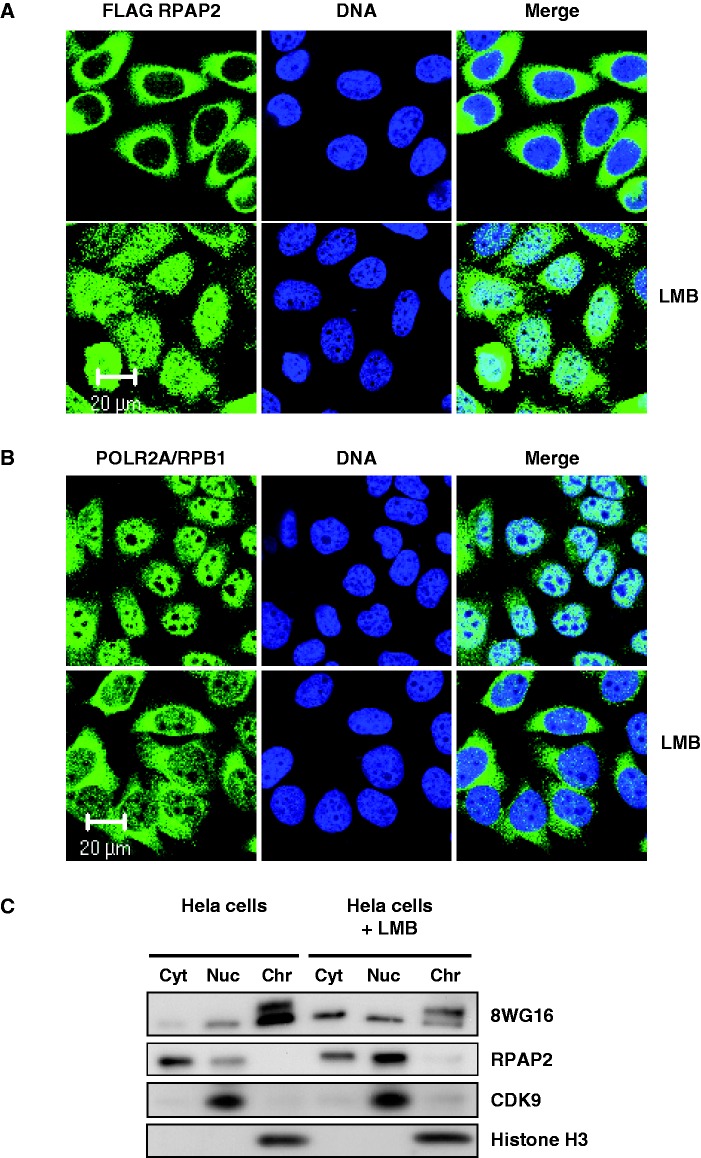
Inhibition of the CRM1/NES nuclear export pathway results in the sequestration of RPAP2 in the nucleus and accumulation of RNAP II in the cytoplasm. (**A**) RPAP2 is a mainly cytoplasmic protein that is re-localized in the nuclear space after cell treatment with the CRM1/NES pharmacological inhibitor LMB. (**B**) The accumulation of RPAP2 in the nuclear space as a result of LMB treatment is paralleled by an accumulation of the largest RNAP II subunit POLR2A/RPB1 in the cytoplasm. In each case, the localization of the DNA is shown (TO-PRO®-3 iodide). In these immunofluorescence experiments, localization of RPAP2 was performed after transfection of FLAG-tagged version of this protein. Localization of endogenous POLR2A/RPB1 used an anti-POLR2A antibody (8WG16). (**C**) Western blot analysis, before or after LMB treatment, was used to monitor RPAP2 and POLR2A/RPB1 in cytoplasmic, nucleoplasmic and chromatin fractions. CDK9 and histone H3 are used as chromatin and nucleoplasmic markers.

Function of the CTD phosphatase RPAP2 during transcription requires that this protein be accessible in the nucleus. Immunofluorescence experiments performed using HeLa cells indicate a mainly cytoplasmic localization for RPAP2 (Figure 1A). However, treatment of the cells with LMB, an inhibitor of nuclear export through the CRM1/NES pathway (35,36), resulted in the accumulation of RPAP2 in the nucleus (Figure 1A), showing that the protein shuttles between the two compartments. LMB treatment also results in the sequestration of RNAP II in the cytoplasm (Figure 1B) [also see (17)]. Western blotting experiments after cell fractionation confirmed the shift in RPAP2 and RNAP II nucleocytoplasmic partitioning as a result of LMB treatment (Figure 1C). Before LMB treatment, RPAP2 is mainly cytoplasmic, whereas it becomes mainly nuclear after treatment.

### Distinct domains of RPAP2 specify nuclear and cytoplasmic localization

**Figure 2.**
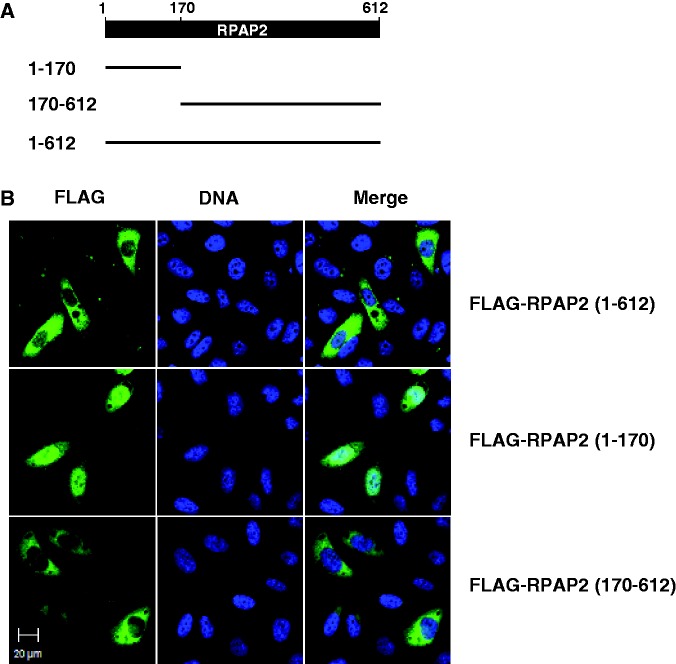
Internal domains of RPAP2 specify nuclear and cytoplasmic localization. (**A**) Representation of RPAP2 fragments used in intracellular localization experiments. (**B**) N-terminal (amino acids 1–170) and C-terminal (amino acids 170–612) fragments having a FLAG tag were transfected in HeLa cells, and an anti-FLAG antibody was used for immunolocalization of the fragments. Localization of the full-length protein (amino acids 1–612) is also shown.

To start defining the mechanism of RPAP2 nucleocytoplasmic partitioning, we transfected fragments of the protein in HeLa cells and determined their intracellular localization of each fragment using immunofluorescence. Figure 2 indicates that an RPAP2 fragment spanning amino acids 1–170 is retained in the nucleus, whereas a fragment spanning from amino acids 170 to 612 accumulates in the cytoplasm. As expected, the exogenous full-length protein is mainly cytoplasmic. These results suggest that a nuclear retention domain is present in the N-terminal region, whereas a dominant cytoplasmic retention domain is located in the C-terminal part of RPAP2. Examination of the primary structure of RPAP2 neither revealed the presence of classical nuclear localization (NLS) nor nuclear export (NES) signals.

### RPAP2 binds to RNAP II through its nuclear retention domain (amino acid 1 and 170)

**Figure 3.**
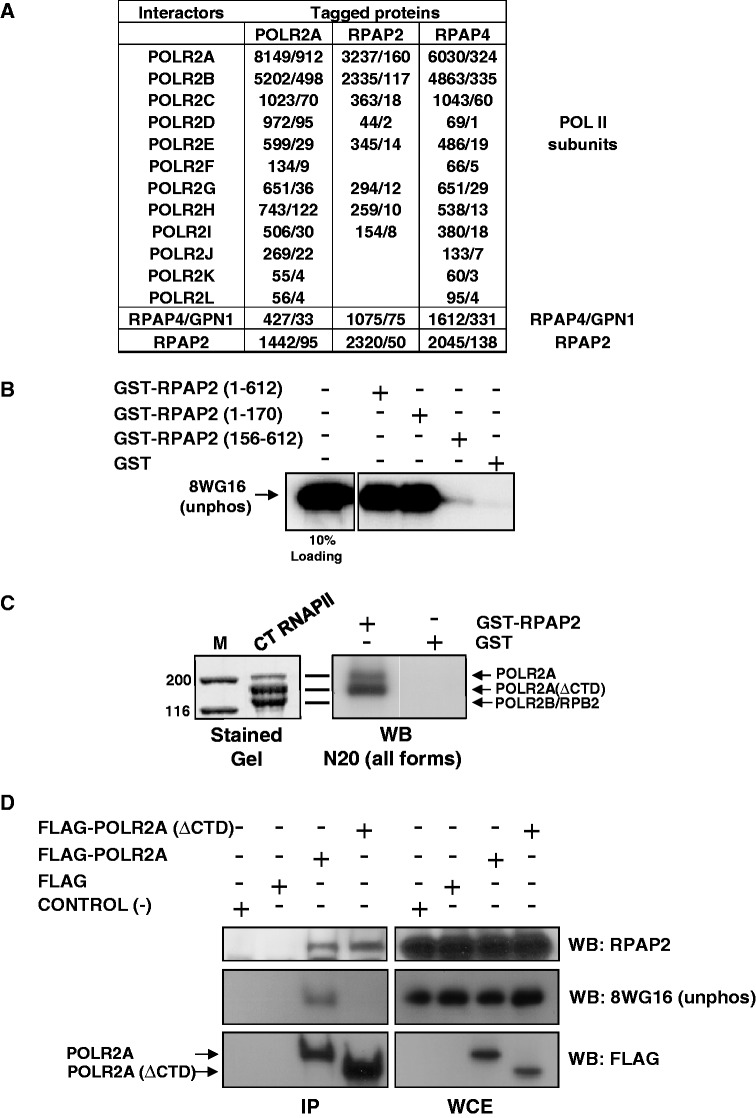
RPAP2 binds directly to RNA polymerase II through the N-terminal (amino acids 1–170) domain. (**A**) Both RPAP4/GPN1 and RPAP2 co-purify with affinity-tagged POLR2A/RPB1 in pull-down experiments. Reversely, RNAP II subunits also co-purify with RPAP4/GPN1 and RPAP2 in similar pull-down experiments, as well as RPAP2 with RPAP4/GPN1 and RPAP4/GPN1 with RPAP2. In each case, mascot scores and then spectral counts are provided. (**B**) *In vitro* GST pull-down experiments were used to show that recombinant RPAP2 binds directly to highly purified calf thymus RNAP II through a domain encompassing its first 170 amino acids, but not to GST used as a control. (**C**) *In vitro* pull-down experiments where GST-tagged RPAP2 or GST alone (control) is mixed with highly purified calf thymus (CT) RNAP II are shown. Glutathione bead are used for the pull-downs, and the N20 anti-POLR2A/RPB1 antibody for protein detection using western blotting (WB). The left panel presents a Coomassie stained sodium dodecyl sulfate gel of highly purified CT RNAP II, showing the IIa (POLR2A) and the IIb (POLR2A(ΔCTD) forms of POLR2A/RPB1 and POLR2B/RPB2. (**D**) *In vivo* immunoprecipitation experiments with an anti-FLAG M2 affinity gel using extracts of HeLa cells where FLAG-tagged POLR2A/RPB1 having a CTD or not (ΔCTD) (or the FLAG alone as a control) have been expressed. Both the immunoprecipitate (IP) and the whole-cell extract (WCE) were probed with 8WG16 (POLR2A/RPB1), RPAP2 and M2 anti-FLAG antibodies.

AP–MS indicates that RPAP2 associates with RNAP II subunits and the GTPase RPAP4/GPN1 in human cell extracts (see Figure 3A and Supplementary Figure S1 for an overall diagram of RNAP and RPAP interactions). To assess whether the RPAP2–RNAP II interaction is direct, we purified recombinant RPAP2 having a GST tag and performed *in vitro* GST pull-down assays using highly purified calf thymus RNAP II. As shown in Figure 3B, RNAP II bound to GST–RPAP2, but not to GST alone used as a control, indicating that RPAP2 makes direct contact with RNAP II. To define the domain of RPAP2 that interacts with RNAP II, we cloned and purified various recombinant RPAP2 fragments with C- and N-terminal deletions and used them *in vitro* GST pull-down experiments. The results indicate that RNAP II binds to a domain of RPAP2 encompassing its first 170 amino acids (Figure 3B).

Because recent articles indicated that both yeast Rtr1 and human RPAP2 bind to and dephosphorylate the POLR2A/RPB1 CTD (32), we asked whether the CTD is required for RPAP2 binding to RNAP II. *In vitro* pull-down experiments where GST-tagged RPAP2 or GST alone (used as a control) is mixed with highly purified calf thymus RNAP II were performed. Preparations of highly purified calf thymus RNAP II contains two predominant forms of the enzyme, the so-called IIa form that contains a hypophosphorylated CTD, and the IIb form that lacks the CTD as a result of proteolysis during purification [Figure 3C, left panel; also see (37)]. The hyperphosphorylated IIo form is also present, but it is 10- to 20-fold less abundant using this purification procedure (38). In our pull-down experiments, GST–RPAP2 bound to both the IIa and IIb forms (as detected using the N20 antibody, which is directed against a peptide located in the N-terminal part of POLR2A/RPB1), an indication that the CTD is not required for binding (Figure 3C, right panel). To confirm this result *in vivo*, we expressed FLAG-tagged POLR2A/RPB1 having a CTD or not (ΔCTD) in HeLa cells and performed a co-immunoprecipitation experiment using an antibody directed against RPAP2. As shown in Figure 3D, the anti-RPAP2 antibody immunoprecipitated both the full-length and the truncated (ΔCTD) POLR2A/RPB1. This result confirms the *in vitro* conclusion that the CTD is not required for efficient binding of RNAP II to RPAP2. Moreover, antibodies directed against various forms of the CTD (N-20, 8WG16, H5 and H14) were successfully used to detect POLR2A in the RPAP2 immunoprecipitate (Supplementary Figure S2), suggesting that modification of the CTD does not significantly affect RPAP2 binding. Of note, these results do not exclude the possibility that RPAP2 also interacts with the RPB1/POLR2A CTD, but rather imply that CTD binding is not essential for the RPAP2–RNAP II interaction.

### RPAP2 binds to the GTPase GPN1/RPAP4 through its cytoplasmic localization domain (amino acid 156–612)

**Figure 4.**
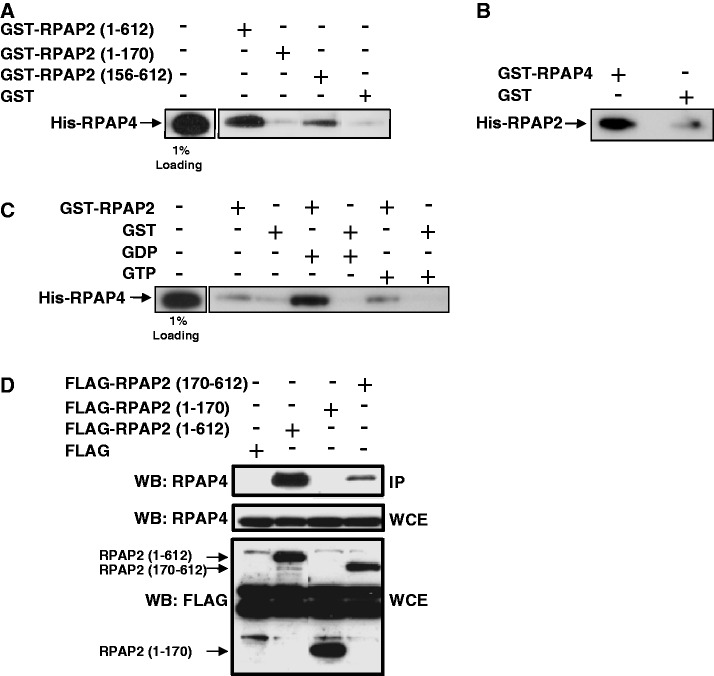
RPAP2 binds directly to RPAP4/GPN1 through the C-terminal (amino acids 156–612) domain. (**A**) Recombinant GST–RPAP2 binds directly to recombinant His–RPAP4/GPN1 through an RPAP2 domain encompassing amino acids 156–612. (**B**) Reversely, recombinant His–RPAP2 binds to recombinant GST–RPAP4. (**C**) The interaction between His–RPAP4 and GST–RPAP2 is increased in the presence of GDP, but not GTP (in the form of non-hydrolysable GTP γ S). In each experiment, a fraction of the input (1% loading) is shown. (**D**) *In vivo* immunoprecipitation experiments with an anti-FLAG M2 affinity gel using extracts of HeLa cells where FLAG-tagged RPAP2 fragments have been expressed. Both the WCE and IP eluates were probed with RPAP4 and M2 anti-FLAG antibodies.

As mentioned in the previous section, the GTPase GPN1/RPAP4 was shown to associate with RPAP2 using AP–MS (Figure 3A). As shown in Figure 4A, we performed *in vitro* pull-down experiments using purified recombinant proteins to assess whether the interaction is direct. His–RPAP4 bound to GST–RPAP2 through a domain encompassing amino acids 156–612. Reversely, His–RPAP2 also bound to GST–RPAP4 (Figure 4B). Notably, binding of RPAP2 to RPAP4/GPN1 is increased in the presence of GDP but not GTP (Figure 4C). Binding of RPAP2 to RPAP4/GPN1 was confirmed using *in vivo* pull-down experiments where tagged RPAP2 fragments were expressed in HeLa cells (Figure 4D). Together, these results indicate that RPAP2 makes direct contact with RPAP4/GPN1 through a domain distinct from its RNAP II-binding domain, and that the RPAP2–RPAP4/GPN1 interaction is strengthened by GDP.

### Silencing of RPAP2 induces the accumulation of RNAP II in the cytoplasm, and silencing of GPN1/RPAP4 leads to retention of RPAP2 in the nucleus

**Figure 5.**
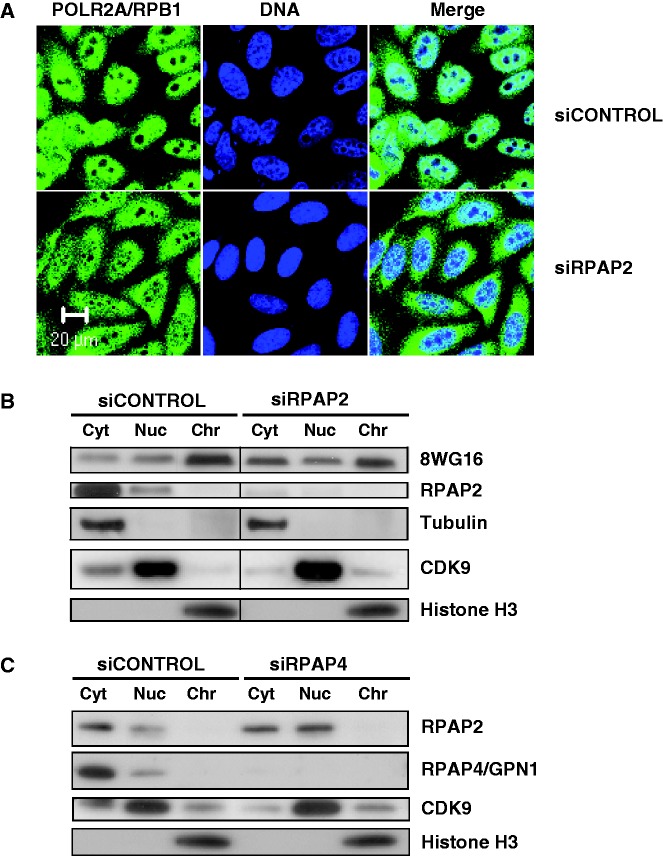
Perturbation of RPAP2 expression leads to the accumulation of RNA polymerase II in the cytoplasm. (**A**) Localization of endogenous POLR2A/RPB1 (8WG16) was determined by immunofluorescence after treatment of HeLa cells with either an siRNA targeting RPAP2 or a control siRNA. In each case, the localization of the DNA is shown (TO-PRO®-3 iodide). (**B**) Western blotting analysis showing the efficiency of RPAP2 silencing in our experiments and its effect on levels of POLR2A/RPB1 (8WG16) in the cytoplasmic, nucleoplasmic and chromatin fractions. CDK9, tubulin and histone H3 are used as cytoplasmic, nucleoplasmic and chromatin markers, respectively. (**C**) Western blotting analysis showing the efficiency of RPAP4 silencing in our experiments and its effect on levels of RPAP2 in the cytoplasmic, nucleoplasmic and chromatin fractions. CDK9 and histone H3 are used as nucleoplasmic and chromatin markers, respectively.

Binding of RPAP2 to RNAP II and GPN1/RPAP4 suggests a possible connection between nucleocytoplasmic shuttling of these proteins. To assess whether RPAP2 is involved in RNAP II nuclear import, we performed siRNA silencing experiments for this protein and monitored the effect on POLR2A/RPB1 localization. As we observed for RPAP4/GPN1 (17), RPAP2 silencing led to a defect in RNAP II nuclear import, as measured through the accumulation of its largest subunit POLR2A/RPB1 in the cytoplasm (Figure 5A). RPAP2 silencing did not affect the intracellular localization of CDK9 (mainly nuclear) and RPAP3 (mainly cytoplasmic) used as controls (Supplementary Figure S3). The efficiency of siRNA silencing was monitored by western blotting, and the effect on POLR2A/RPB1 localization was confirmed biochemically (Figure 5B). Because we were only able to detect cytoplasmic accumulation of POLR2A/RPB1 using the 8WG16 antibody in our immunofluorescence studies, and not antibodies directed against phosphorylated forms of the CTD (H14 and CC3), which are nuclear, we concluded that the unphosphorylated POLR2A/RPB1 specifically accumulates in the cytoplasm in the absence of RPAP2 (Supplementary Figure S4). Notably, RPAP2 silencing was not found to affect the normal mainly cytoplasmic localization of GPN1/RPAP4 (Supplementary Figure S5). Together with our data on binding and localization domains, these results indicate that RPAP2 is required for nuclear import of RNAP II by associating with the enzyme through its N-terminal domain spanning amino acids 1–170.

We next proceeded to assess the role of GPN1/RPAP4 in nucleocytoplasmic shuttling of RPAP2. As we have shown previously that GPN1/RPAP4 is required for nuclear import of RNAP II (17), we wanted to know whether this factor is also involved in nuclear import of RPAP2. To our surprise, the results indicate that GPN1/RPAP4 silencing induces nuclear retention of RPAP2 as determined by western blotting after cell fractionation (Figure 5C). Together with our data on binding and localization domains, this result indicates that RPAP2 returns to the cytoplasm in association with the GTPase GPN1/RPAP4, whose nuclear export in CRM1 dependent (39).

## DISCUSSION

**Figure 6.**
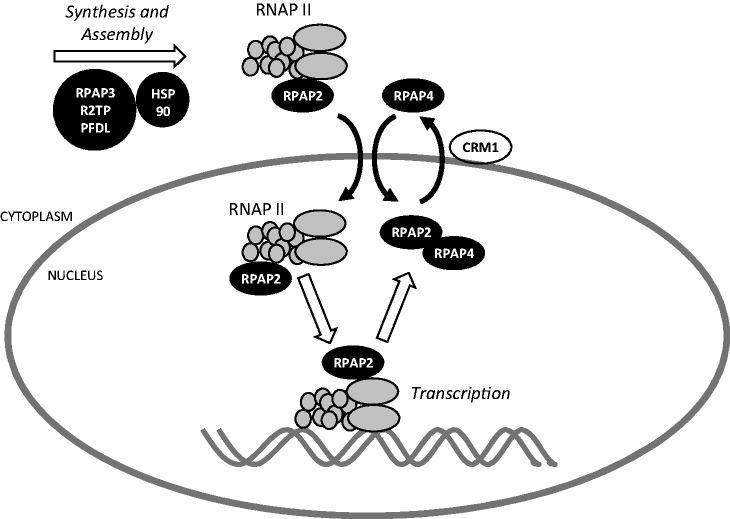
Model for nuclear import of RNAP II coupled with nucleocytoplasmic shuttling of RPAP2 and RPAP4/GPN1. Subunits of RNAP II are synthesized in the cytoplasm where enzyme assembly proceeds through the action of HSP90 and the RPAP3/R2TP/PFDL complex, which acts as a HSP90 cofactor. RNAP II is imported to the nucleus with the CTD phosphatase RPAP2, the latest being required for RNAP II nuclear import. Whether RNAP II is imported to the nucleus in the form of the fully assembled enzyme or sub-complexes is unclear. RPAP2 returns to the cytoplasm by associating with the GTPase RPAP4/GPN1. Whether RPAP2 dissociates from RNAP II immediately after import or later after the transcription reaction is terminated is not known. In this model, RPAP2 regulates transcription both by acting as an RNAP II CTD phosphatase during the transcription reaction and as an RNAP II nuclear import factor.

Biogenesis of RNAP II is a poorly understood process that recently gained in popularity through the identification of factors involved in nuclear import and assembly of this key molecular machine. Through affinity purification coupled with mass spectrometry, Jeronimo *et al.* (19) have characterized a network of interactions involving RNAP II subunits in the soluble human cell fraction. Functional and biochemical analyses of these proteins now lead to the emergence of a model for describing this central cellular process (Figure 6). According to this model, assembly of RNAP II from its 12 subunits takes place in the cytoplasm through the action of the molecular chaperone HSP90 and co-chaperone RPAP3. RPAP3 is part of an 11-subunit complex, called the RPAP3/R2TP/PFDL complex, that contains other chaperones and co-chaperones (Supplementary Figure S1). Nuclear import of pre-assembled RNAP II requires at least two specific factors, GPN1/RPAP4 and RPAP2. The GTPase GPN1/RPAP4 shuttles between the cytoplasm and the nucleus through the action of a classical NES (39). RPAP2 is imported to the nucleus in association with RNAP II, an association that requires an RPAP2 domain encompassing its first 170 residues. Requirement of RPAP2 for RNAP II nuclear import is supported by finding that RPAP2 silencing generates an accumulation of RNAP II in the cytoplasmic space. Notably, the RPAP2 N-terminal domain spanning from amino acids 1–170 specifies nuclear localization of this protein, most likely by allowing RPAP2 to associate with RNAP II, and cannot return to the cytoplasm, as it lacks the RPAP4/GPN1-binding domain (amino acids 156–612).

Silencing of the GTPase GPN1/RPAP4 also leads to abnormal cytoplasmic localization of RNAP II. Our results demonstrate that RPAP4/GPN1 binds directly to RPAP2 through a domain spanning amino acids 156–612, an interaction that is strengthened by the presence of GDP. Interestingly, silencing of RPAP4/GPN1 results in nuclear retention of RPAP2, suggesting that RPAP4/GPN1 is required for nuclear export of RPAP2. The model in Figure 6 accounts for these findings. According to the model, RPAP2 binds directly to RNAP II through its N-terminal domain 1–170 in the cytoplasm, and both proteins are imported together to the nucleus. In the nucleus, RNAP II is released and RPAP4/GPN1 returns to the cytoplasm in association with RPAP2. What regulates association/dissociation of RPAP4/GPN1 and RPAP2 from RNAP II has not yet been determined. We speculate that other regulatory factors, such as the CTD-interacting domain (CID) proteins or Iwr1 may play a role. As it has been shown that the function of RPAP4/GPN1 is coupled with microtubule assembly (17), one possibility is that microtubules are involved in the coupled processes of RNAP II nuclear import and RPAP2 nucleocytoplasmic shuttling. This model poses that assembly and nuclear import of the enzyme are tightly coupled, a notion that is supported by the interaction network formed by these proteins in the soluble cell fraction (Supplementary Figure S1). Notably, silencing of RPAP3 was found to have a similar effect as RPAP2 and RPAP4/GPN1 silencing, also leading to accumulation of RNAP II subunits in the cytoplasm (Forget *et al.*, in press). Of note, and similarly to what has been obtained for RPAP4/GPN1 (17), silencing of RPAP2 causes accumulation of the largest RNAPII subunits POLR2A and POLR2B in the cytoplasm, whereas the smaller subunits can reach the nuclear space (data not shown). It is unclear at this point whether this observation is the result of RNAP II assembly defects caused by aberrant nuclear import or an indication that sub-complexes, whose existence has been alluded to in other reports (26,40), are imported independently to the nucleus where final assembly could take place.

We provide evidence that RPAP2 shuttles between the nucleus and the cytoplasm in a mechanism that is tightly coupled with nuclear import of RNAP II. Indeed, we identified distinct regions of RPAP2 that interact with RNAP II (amino acids 1–170) and the RNAP II import factor GPN1/RPAP4 (amino acids 156–612), respectively. Silencing of RPAP2 and GPN1/RPAP4, and inhibition of RPAP2 and GPN1/RPAP4 shuttling through the use of LMB, both result in the accumulation of RNAP II subunits in the cytoplasm. Interestingly, we submitted the RPAP2 sequence to tertiary structural prediction using the I-TASSER protein threading software (41). I-TASSER outputted two putative structures with equal C-scores, one resembling a karyopherin and the other a helicase. We, therefore, repeated the analysis for all mammalian RPAP2 orthologs currently available in the SWISS-PROT database (bovine, mouse, rat and orangutan). The helicase-like fold had relatively little support in these orthologs, but the vast majority of the candidate high-scoring structures (C-score > −1.5, template modelling (TM)-score > 0.17) obtained from orthologous sequences had a karyopherin-like structure. Indeed, those structures showed high structural similarity to human Karyopherin β 2 (TM-score = 0.949), human importin β 1 (TM-score = 0.907) and human importin 13 (TM-score = 0.646) (data not shown). This computational analysis suggests that RPAP2 may act as an importin-like protein during RNAP II nuclear import (Supplementary Figure S6). To this effect, stimulation of RPAP2–GPN1/RPAP4 binding by GDP is reminiscent of the case of other importins that form a complex with another GTPase, Ran, during import of various cargoes (36,42). We do not have evidence at this stage that the RPAPs participate in nuclear import of other proteins and may, therefore, be specific to RNAP subunits in mammals.

In a recent article, Murphy and colleagues (32) provided evidence for direct binding of RPAP2 to the POLR2A/RPB1 CTD. These results are in agreement with those of Washburn and colleagues (33) on the yeast homolog of RPAP2, Rtr1. Both groups also presented evidence that human RPAP2 and yeast Rtr1 have a phosphatase activity that targets the POLR2A/RPB1 CTD. Although our results do not directly address the putative CTD phosphatase function of RPAP2 at specific gene loci during transcription, they demonstrate a function of RPAP2 in RNAP II biogenesis (i.e. nuclear import and/or assembly) and indicate that the RNAP II–RPAP2 interaction does not necessitate the presence of the POLR2A/RPB1 CTD. Notably, and in support to our conclusions, the structure of *Lactobacillus casei* Rtr1 indicates that the protein neither does have a CTD-binding pocket nor does it phosphorylate the CTD (43).

Most importantly, our results propose a dual mechanism of gene regulation by the CTD phosphatase RPAP2. In addition to being able to associate with transcribing RNAP II molecules to regulate CTD phosphorylation and polymerase transcriptional activity, RPAP2 itself shuttles between the cytoplasm and the nucleus in a process that is required for nuclear import of RNAP II. We do not believe at this point that CTD phosphorylation is indeed involved in RNAP II nuclear import, but rather that association of RNAP II and RPAP2 participates in the biogenesis of an RNAP II complex poised to transcriptional activation and promoter recruitment.

## CONCLUSIONS

Since the discovery of RNA polymerase II more than 3 decades ago, many regulatory factors and mechanisms have been uncovered to account for the extensive modulation of gene expression during cell growth, differentiation and response to various stimuli. These mechanisms were shown to affect various stages of the transcription reaction, either by acting directly on the polymerase and its accessory machinery or by modulating the architecture and accessibility of the chromatin template. In this article, we reported on the discovery of an unprecedented regulatory mechanism where nucleocytoplasmic shuttling of the CTD phosphatase RPAP2 is required for nuclear import RNA polymerase II. Because RPAP2 was previously shown to modulate the activity of polymerase molecules through dephosphorylation of the CTD domain, this novel finding indicates that RPAP2 has the ability to regulate transcription at a second level, by promoting nuclear import of newly assembled polymerases, thereby controlling RNA polymerase II availability for transcription.

## SUPPLEMENTARY DATA

Supplementary Data are available at NAR Online: Supplementary Figures 1–6.

## FUNDING

Canadian Institutes of Health Research (CIHR); Fonds de recherche du Québec - Santé (FRQS). A.A.L. and M.L.A. hold studentships from the FRQS and the Natural Sciences and Engineering Research Council of Canada (NSERC). Funding for open access charge: Fonds de recherche du Québec - Santé (FRQS).

*Conflict of interest statement.* None declared.

## Supplementary Material

Supplementary Data
