# Supplementary material for: Nuclear import of RNA polymerase II is coupled with nucleocytoplasmic shuttling of the RNA polymerase II-associated protein 2
Source: Nucleic Acids Res. 2013 May 30;41(14):6881–91. doi: 10.1093/nar/gkt455 (PMC3737550; doi:10.1093/nar/gkt455)
Supplement: Supplementary Data [file supp_41_14_6881__index.html]

Nuclear import of RNA polymerase II is coupled with nucleocytoplasmic shuttling of the RNA polymerase II-associated protein 2 — Nuclear import of RNA polymerase II is coupled with nucleocytoplasmic shuttling of the RNA polymerase II-associated protein 2 — Supplementary Data 

# Nuclear import of RNA polymerase II is coupled with nucleocytoplasmic shuttling of the RNA polymerase II-associated protein 2

## Supplementary Data

files

**Files in this Data Supplement:**

- Supplementary Data - pdf file
