# Supplementary material for: Nuclear import of RNA polymerase II is coupled with nucleocytoplasmic shuttling of the RNA polymerase II-associated protein 2
Source: Nucleic Acids Res. 2013 May 30;41(14):6881–91. doi: 10.1093/nar/gkt455 (PMC3737550; doi:10.1093/nar/gkt455)
Supplement: Supplementary Data [file supp_gkt455_nar-00735-r-2013-File008.pdf]

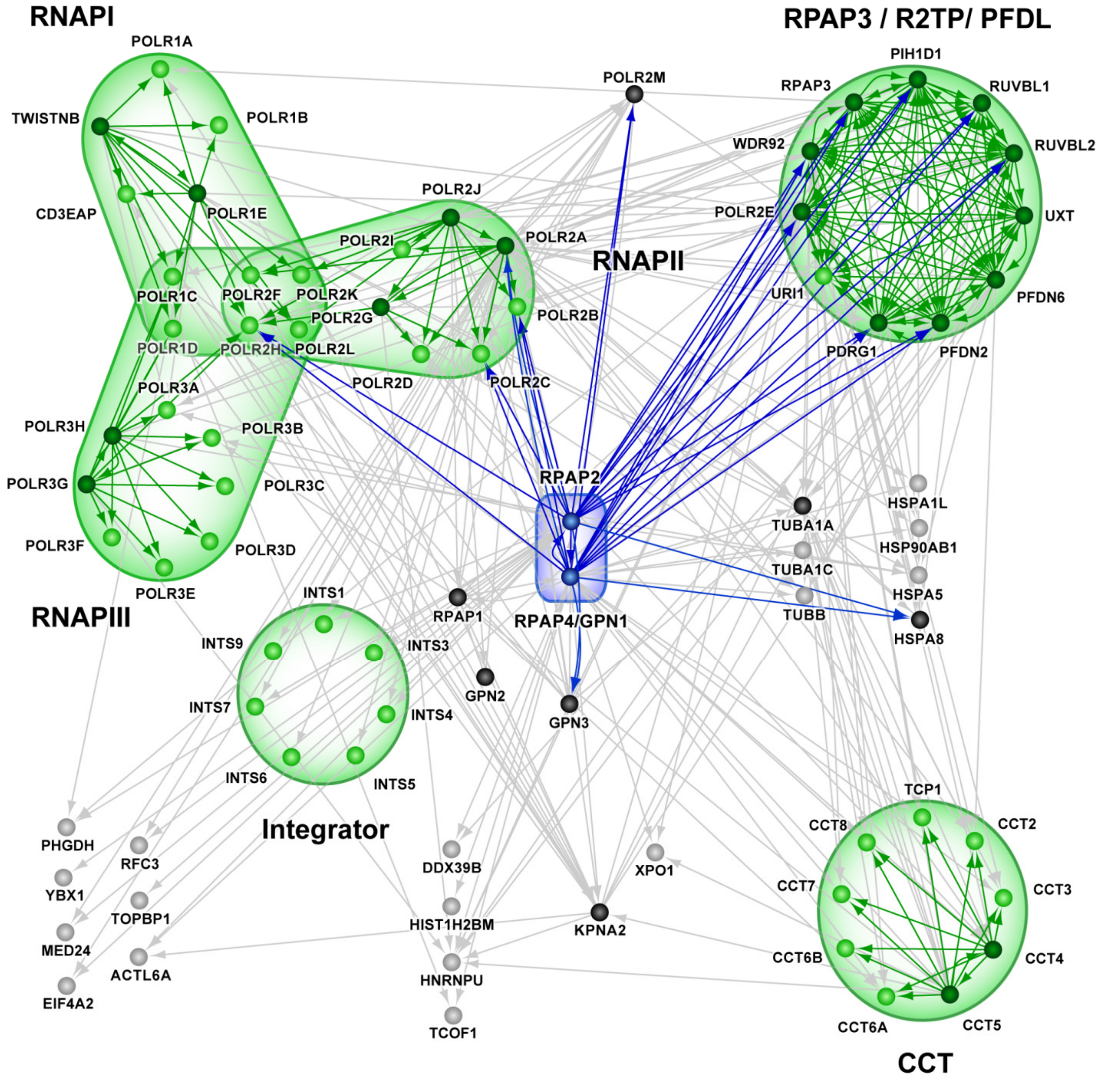

Figure S1. Diagram of high confidence interactions involving nuclear RNA polymerase and RPAP subunits. This diagram summarizes protein-protein interactions identified experimentally by our laboratory using affinity purification coupled with mass spectrometry (AP-MS), and shows the central position of RPAP2 and RPAP4/GPN1 in the network. The dataset and score threshold used here are as in Ref. 17.

Figure S1

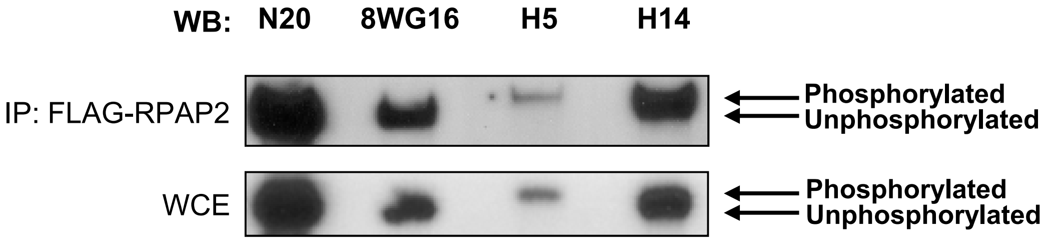

Figure S2. Phosphorylation of the POLR2A CTD does not significantly affect binding of RNA polymerase II to FLAG-RPAP2. Antibodies that preferentially recognize various phosphorylation states of the CTD were used to analyse the FLAG-RPAP2 immunoprecipitate. The preferred specificity of the antibodies is: N20 = all forms, 8WG16 = unphosphorylated, H5 = Ser2 phosphorylation, and H14 = Ser5 phosphorylation.

Figure S2

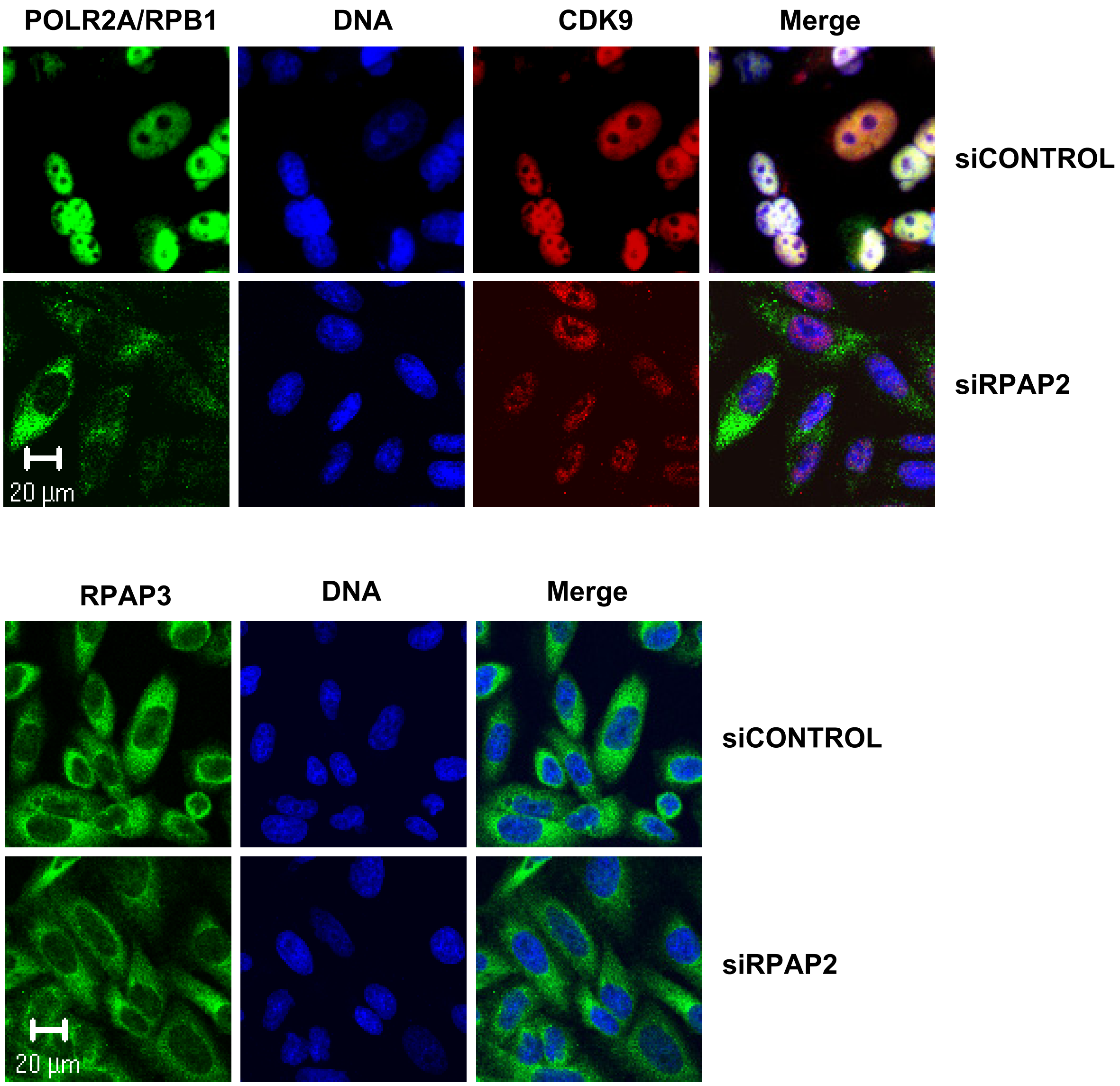

Figure S3. RPAP2 silencing does not affect the nuclear localization of CDK9 and the cytoplasmic localization of RPAP3.

Figure S3

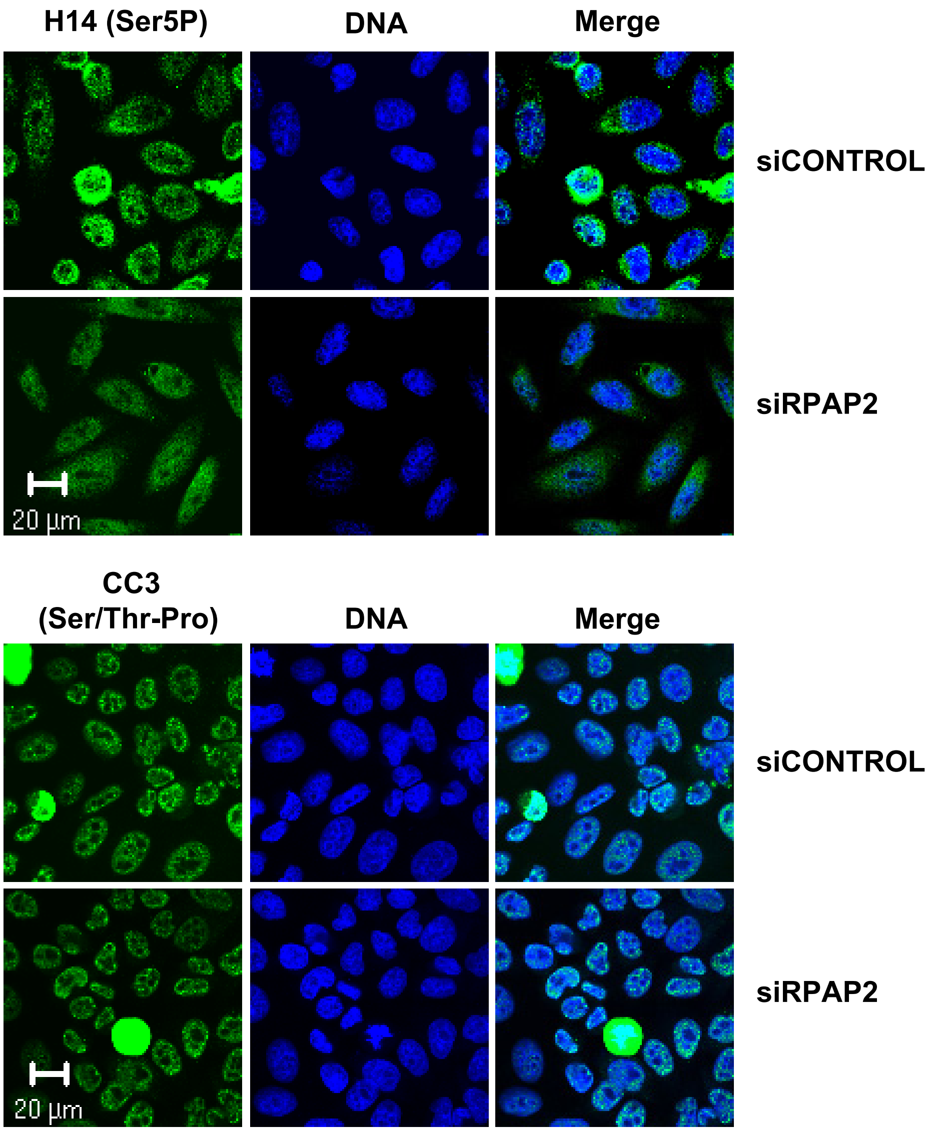

Figure S4. RPAP2 silencing does not affect the nuclear localization of phosphorylated forms of RNA polymerase II (POLR2A) as detected by immunofluorecence using the H14 (Ser5-P) and CC3 (Ser/Thr-Pro-P) antibodies.

Figure S4

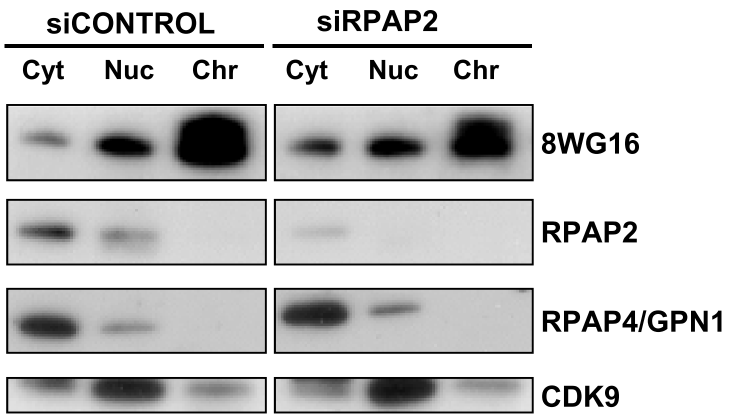

Figure S5. RPAP2 silencing does not affect the cytoplasmic localization of RPAP4/GPN1 as detected by western blotting following cell fractionation into cytoplasmic, nucleoplasmic, and chromatin fractions.

Figure S5

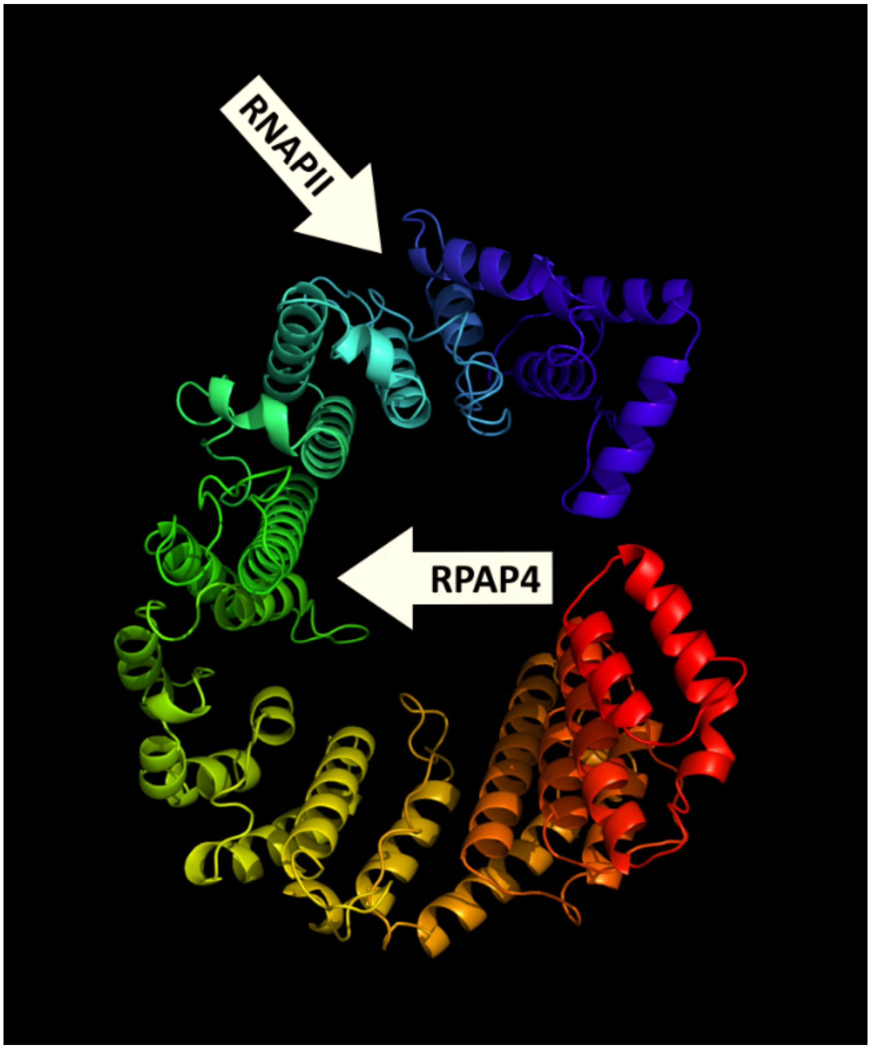

Figure S6. Model of RPAP2 structure predicted by the I-TASSER software. Binding domains of RNAP II and RPAP4/GPN1 are indicated.

Figure S6
